# Supplementary material for: Cross-cultural adaptation and psychometric evaluation of the Yoruba version of Oswestry disability index
Source: PLoS One. 2020 Jan 8;15(1):e0221138. doi: 10.1371/journal.pone.0221138 (PMC6949003; doi:10.1371/journal.pone.0221138)
Supplement: S1 Text — (DOCX) [file pone.0221138.s003.docx]

**Ìgbéléwòn Bèbèré Èyìn Dídùn ti Oswestry**

Jòwó fi ìwòn bí ìrora re se le tó hàn nípa yíyí òdo sí orí náńbà kan nísàlè

Kò sí ìrora [0] [1] [2] [3] [4] [5] [6] [7] [8] [9] [10] kò se é mú móra

Orúko ____________________________ Déètì ______________________

Ìwé ìbéèrè yìí wà láti pèsè àlàyé fún dókítà nípa bí ìrora eyin re se ń kópa nínú ìgbé-ayé òòjó re. Jòwó fi àmì sí **NÓŃBÀ KAN** soso ni abala kòòkan, èyí tí ó bá ìsòro re mu jù lo. A m ò pé o se é se kí gbólóhùn méjì bá ohun tó ńse ó mu ní abala kan, èyí tí ó bá kàn ó jù lo ni kí o fi àmì sí.

**Abala kìn-ín-ní - Bí ìrora se le tó**

(0) Ìrora náà máa ń wá, ó máa ń lo ni sùgbon kò le

(1) Ìrora náà kò le sùgbón kìí yàtò púpò

(2) Ìrora náà máa ń wá, ó máa ńlo ni sùgbón kò kojá àfaradà

(3) Ìrora náà kò kojá àfaradà kìí sì yàtò púpò

(4) Ìrora náà máa ń wá, ó máa ń lo ni, ó sì le púpò

(5) Ìrora náà le púpò, kìí sìí yàtò púpò

**Abala Kejì - Ìtójú ara eni (fifo nnkan, ara múmú, abbl)**

(0) N kò ní láti yí ònà tí mò ń gbà fo nnkan àti ònà tí mò ń gbà múra padà kí n lè yàgò fún ìrora.

(1) N kìí sáábà yí ònà tí mò ń gba fo nnkan tàbí tí mò ń gbà múra padà bí ó tilè jé pé ó máa ń

mú ìrora díe dání.

(2) Nnkan fifò àti ara mímú túbò máa ń mú kí ìrora náà pò síi ni sùgbón m ò ń gbìyànjú láti má

yí bí mo se ń se é padà.

(3) Nnkan fífò àti ara mímú máa ń mú kí ìrora náà pò síi ni, mo sì rí pé ó ye kí n yí bí mo se

ń se wón padà.

(4) Nítorí ìrora náà, n kò lè fo nnkan dí è tàbí múra láì rí ìrànlówó

(5) Nítorí ìrora náà, n kò lè fo nnkan kan, tàbí múra rárá láì rí ìrànlówó

**Abala Kéta –Gbígbé Nnkan**

(0) Mo lè gbé ohun tó wúwo láì sí àfikún ìrora

(1) Mo le gbé ohun tó wúwo sùgbón ó máa fún mi ní àfikún ìrora.

(2) Ìrora máa dí mi lówó láti gbé erù tó wúwo kúrò nílè

(3) Ìrora máa ń di mi lówó láti gbé erù tó wúwo kúrò nílè sùgbón mo lè gbé e bí ó bá wà ni

ipò tó rò mí lórùn fún àpeere, lórí tábìlì

(4) Ìrora máa ń di mi lówó láti gbé erù tó wúwo sùgbón mo lè gbìyànjú láti gbè èyí tó fúyé

tàbí tí kò wúwo púpò bí wón bá wà ní ipò tó rò mí lórùn.

(5) Erù tó fúyé nìkan ni mo lè gbé tó pò jù.

**Abala Kérin - Ìrìn rínrìn**

(0) N kò ní ìrora nípa ìrìn rínrìn

(1) Mo ní ìrora tó je mó ìrìn sùgbón kìí pò síi bí ibi tí mò ń lo bá se jìnnà sí

(2) Mo lè rìn ju máìlì kan lo láì jé pé ìrora náà pò síi

(3) N kò lè rin ju ìlàjì máìlì lo láì sí àfikún ìrora

(4) Nkò lè rìn ju ìdámérin máìlì kan láì sí àfikún ìrora

(5) N kò lè rìn rárá láì sí ìrora

**Abala Kárùn-ún–Ipò ìjókòó**

(0) Mo lè jókòó lórí àga kágaga bí mo bá se fé

(1) Orí àga tí mo féràn jù ni mo le jókòó lé bí mo bá se fé

(2) Ìrora máa ń di mi lówó láti jókòó ko já wákàtí kan

(3) Ìrora máa ń di mi lówó láti jókòó ko já ìdàjì wákàtí kan

(4) Ìrora máa ń dí mi lówó láti jókòó kojá ìséjú méwàá.

(5) Mo máa ń yàgò fún jíjókòó nítorí lésè kesè ló máa ń fi kún ìrora mi

**Abala Kefà–Ìnàró**

(0) Mo lè nàró fún iye àkókò tí mo bá fé lá ìsí ìrora

(1) Mo máa ń ní ìrora pèlú ìnàwó sùgbón kìí pò sí pèlú àkókó

(2) N kò lè nàró kojá wákàtí kan láì sí àfikún ìrora

(3) N kò lè nàró kojá ìdajì wákàtí láì sí àfikún ìrora

(4) N kò lè nàró kojá ìséjú méwàá láì sí àfikún ìrora

(5) Mo máa ń sá fún ìnàwó nítorí lésè kesè ló máa ń fi kún ìrora mi

**Abala Kéje–Oorun sísùn**

(0) Ara kìí ro mí lórí béèdì

(1) Ara máa ń ro mí lórí béèdì sùgbón kò dí mi lówó lá ti sùn

(2) Nítorí ìrora, oorun alé mi máa ń dínkù pèlú bí i ìdámérin

(3) Nítorí ìrora, oorun alé mi ti dínkù sí bí ìdajì

(4) Nítorí ìrora, oorun alé mi ti dínkù sí bí ìdá kan nínú ìda mérin

(5) Ìrora máa ń di mi lówó láti sùn rárá ni

**Abala Kéjo-Ìgbé-ayé ní àwùjo**

(0) Ìgbé-ayé mi láwùjo kò fún mi nì ìròra

(1) Ìgbé-ayé mi láwùjo dára sùgbón ó máa ń fi kún ìrora mi

(2) Ìrora kò ní ipa kan pàtó lórí ìgbé-ayé mi láwùjo ju pé ó ń di mi lónà láti se àwon

nnkan tó wù mí to sì la agbára lo, fún àpeere, ijó jíjó abbl.

(3) Ìrora ti dí ìgbé-ayé mi láwùjo lówó, n kò sì sáábà máa ń jáde mó

(4) Ìrora ti dé mi mólé

(5) Kò sí igbé-ayé kankan fún mi láwùjo nítorí ìrora

**Abala Késàn-án–Rínrin ìrìnàjò**

(0) N kìí ní ìrora bí mo bá ń rìnrìn-àjò

(1) Mo máa ń ní ìrora díè bí mo bá ń rìnrìn-àjò sùgbón kò sí èyí tó mú un búni síi

(2) Mo máa ń ni àfikún wòra bí mo bá ń rìnrìn-àjò sùgbón kò fi ipá mú mi láti wà irúfé ònà

mìíràn láti rìnrìn-àjò

(3) Mo máa ń ni àfikún ìrora bí mo bá ń rìnrìn-àjò tó sì máa ń fipá mú mi láti wá irúfé ònà

mìíràn láti rìnrìn-àjò

(4) Ìrora ń di mi lówó láti máa rìnrìn-àjò tó bá ti kojá ìdajì wákàtí. Àwon tó se pàtàkì ni mò ń rìn.

(5) Ìrora dí mi lówó gbogbo ìrìn-àjò

**Abala Kéwàá–Bí ìwòn ìrora se ń yípadà**

(0) Ìrora mi ń dínkù jojo

(1) Ìrora mi máa ń lo, ó máa n bò ní sùgbón ó ń dínkù jojo

(2) Ìrora mi ń dínkù jojo sùgbón ó ń ló ra láti dínkù

(3) Ìrora mi kò dínkù béè ni kò burú síi

(4) Ìrora mi ń pele síi díè díè

(5) Ìrora mi ń pele síi léraléra ni
