# Supplementary material for: Cross-cultural adaptation and psychometric evaluation of the Yoruba version of Oswestry disability index
Source: PLoS One. 2020 Jan 8;15(1):e0221138. doi: 10.1371/journal.pone.0221138 (PMC6949003; doi:10.1371/journal.pone.0221138)
Supplement: Attachment — Submitted filename: plos.docx [file pone.0221138.s004.docx]

Cross-cultural adaptation and psychometric evaluation of the Yoruba version of
Oswestry disability index

Thank you for the opportunity to review the manuscript. I reviewed this manuscript carefully with a great interest. I respectfully provided my comments below

Methods

1: In section methods no description for validation( face, content, construct(EFA and CFA) and convergent) Please give more details about that.

2: Where did the 280150 samples for EFA and CFA come from? Did the authors do the power analysis for the sample sizes?

3: The choice of PCA to evaluate the factor structure of the ODI-Y appears somewhat rudimentary given the increasingly sophisticated statistically methods that have been used to evaluate the scale since the publication of the original article. It would be good to know why the author has chosen to use PCA rather than using structural equation models which have become much more commonly used to test the psychometric properties of scales.

4: What rotation model of EFA was used?

5: .Say more about the sample of 86 who completed the questionnaire twice. Who were they?

Data Analysis

1:Please report first the result validity next reliability

2 The values for RMSEA is higher and not adequate. Its cut off value is 0.08 according to Bentler & Hu

3: GFI= 0.87; AGFI=0.85: These values should at least be higher than 0.90. The model has inadequate model of fit

4: Lastly, factors should be retained using Parallel Analysis. Scree plot and Eigen values are traditional methods

The discussion part of this paper is not good. It would be better if you could write this part focusing on the results from the study
