# Supplementary material for: Cross-cultural adaptation and psychometric evaluation of the Yoruba version of Oswestry disability index
Source: PLoS One. 2020 Jan 8;15(1):e0221138. doi: 10.1371/journal.pone.0221138 (PMC6949003; doi:10.1371/journal.pone.0221138)
Supplement: Attachment — Submitted filename: plos.docx [file pone.0221138.s005.docx]

Dear Editor,

We were pleased to have an opportunity to revise our manuscript entitled
Cross-cultural adaptation and psychometric evaluation of the Yoruba version of Oswestry disability index (PONE-D-19-21244).

We have carefully considered reviewers’ comments and suggestions. As instructed, we have attempted to succinctly explain changes made in reaction to all comments. Our point by point response to the comments of the academic editor and reviewers has been labeled appropriately. We have also included a marked-up copy of our manuscript that highlights changes made to the original version as well as an unmarked version of your revised paper.

The responses to the concerns raised by reviewers are below and are coded as follows: a) Comments from editors or reviewers are shown as **bold***;* b) Our responses are shown as text. The reviewers’ comments were very helpful overall, and we are appreciative of such constructive feedback on our original submission. After addressing the issues raised, we feel the quality of the paper is much improved.

Yours Sincerely

Idowu OA

(On behalf of Authors)

**Journal Requirements as requested by academic editor**:

**1. When submitting your revision, we need you to address these additional requirements. Please ensure that your manuscript meets PLOS ONE's style requirements, including those for file naming. The PLOS ONE style templates can be found at** [**http://www.journals.plos.org/plosone/s/file?id=wjVg/PLOSOne_formatting_sample_main_body.pdf**](http://www.journals.plos.org/plosone/s/file?id=wjVg/PLOSOne_formatting_sample_main_body.pdf) **and** [**http://www.journals.plos.org/plosone/s/file?id=ba62/PLOSOne_formatting_sample_title_authors_affiliations.pdf**](http://www.journals.plos.org/plosone/s/file?id=ba62/PLOSOne_formatting_sample_title_authors_affiliations.pdf)

A: We have addressed this to the best of our ability. Thank you very much.

**2.  Please include captions for your Supporting Information files at the end of your manuscript, and update any in-text citations to match accordingly. Please see our Supporting Information guidelines for more information:** [**http://journals.plos.org/plosone/s/supporting-information**](http://journals.plos.org/plosone/s/supporting-information)**.**

A: We included captions for our supporting information and updated all in-text citations to match.

**3. Please carefully proofread your manuscript for typographical errors. For example, on page 8 “However, no less 50 participants be considered adequate…” should be “However, no less than 50 participants is considered adequate…”**

A: We have proofread our manuscript and corrected all the typographical errors. Thank you.

4. **Please state in your methods section the participant recruitment date**

.A: The participant recruitment date (10/12/2015 and 10/01/2016) has been included in the methods section) (Page 5, line 109)

**5. Please state the participants’ age range (in addition to their mean age).**

A: The age range of the participants (35-70 years) has been included (Page 11, line 250).

**6. Please amend your current ethics statement and methods section to address the following concerns:   a) Did participants provide their written or verbal informed consent to participate in this study?  b) If consent was verbal, please explain:  i) Why was written consent not obtained?   ii) How did you record/document participant consent?  iii) Did the ethics committees/IRBs approve this consent procedure?**

A: Thank you very much for highlighting this omission in our manuscript. The participants provided their written informed consent. The consent procedure was approved by the ethics committee/IRB (Page 5, line 110).

**7. Please amend the manuscript submission data (via Edit Submission) to include author Ajibola Babatunde Oladiran.**

A: This has been done. Thank you very much for pointing this out.


[Note: HTML markup is below. Please do not edit.]

Reviewers' comments:

Reviewer's Responses to Questions

**Comments to the Author**

1. Is the manuscript technically sound, and do the data support the conclusions?

The manuscript must describe a technically sound piece of scientific research with data that supports the conclusions. Experiments must have been conducted rigorously, with appropriate controls, replication, and sample sizes. The conclusions must be drawn appropriately based on the data presented.

Reviewer #1: Partly

Reviewer #2: Partly

2. Has the statistical analysis been performed appropriately and rigorously?

Reviewer #1: Yes

Reviewer #2: No

3. Have the authors made all data underlying the findings in their manuscript fully available?

The [PLOS Data policy](http://www.plosone.org/static/policies.action#sharing) requires authors to make all data underlying the findings described in their manuscript fully available without restriction, with rare exception (please refer to the Data Availability Statement in the manuscript PDF file). The data should be provided as part of the manuscript or its supporting information, or deposited to a public repository. For example, in addition to summary statistics, the data points behind means, medians and variance measures should be available. If there are restrictions on publicly sharing data—e.g. participant privacy or use of data from a third party—those must be specified.

Reviewer #1: Yes

Reviewer #2: Yes

4. Is the manuscript presented in an intelligible fashion and written in standard English?

PLOS ONE does not copyedit accepted manuscripts, so the language in submitted articles must be clear, correct, and unambiguous. Any typographical or grammatical errors should be corrected at revision, so please note any specific errors here.

Reviewer #1: Yes

Reviewer #2: Yes

5. Review Comments to the Author

Please use the space provided to explain your answers to the questions above. You may also include additional comments for the author, including concerns about dual publication, research ethics, or publication ethics. (Please upload your review as an attachment if it exceeds 20,000 characters)

Reviewer #1: Cross-cultural adaptation and psychometric evaluation of the Yoruba version of Oswestry disability index
Thank you for the opportunity to review the manuscript. I reviewed this manuscript carefully with a great interest. I respectfully provided my comments below

Methods
**1: In methods section no description for validation (face, content, construct (EFA and CFA) and convergent) are provided. Please give more details about that.**

A: Thank you very much for pointing our attention to this; we have included more details about the EFA and convergent validity. We have had to remove the CFA following the suggestion of the second reviewer (Page 9, Line 205- page 10 Line 224).

2: **Where did the 280150 samples for EFA and CFA come from? Did the authors do the power analysis for the sample sizes?**

A: We didn’t do a power analysis. However, we adopted a minimum sample size for the EFA.

**3: The choice of PCA to evaluate the factor structure of the ODI-Y appears somewhat rudimentary given the increasingly sophisticated statistically methods that have been used to evaluate the scale since the publication of the original article. It would be good to know why the author has chosen to use PCA rather than using structural equation models which have become much more commonly used to test the psychometric properties of scales.**

A: Thank you very much for enlightening us about the superiority of structural equation modelling over PCA. Our sample was less than the 200 minimum sample size for the CFA and SEM. We had to use the PCA which our sample was adequate for. We hope to explore this suggestion subsequently.

4: What rotation model of EFA was used?

A: We have included this in the manuscript (Promax rotation): Page 10, line 220)

**5: .Say more about the sample of 86 who completed the questionnaire twice. Who were they?**

A: We have included the mean age and gender of participants who took part in the retest phase “(55.9±11.0 years; 54.7% female)” (Page 9, line 195).

Data Analysis
1:**Please report first the result validity next reliability**

A: This had been done, thank you ( Page 12, lines 260-267)

**2 The values for RMSEA is higher and not adequate. Its cut off value is 0.08 according to Bentler & Hu
3: GFI= 0.87; AGFI=0.85: These values should at least be higher than 0.90. The model has inadequate model of fit**

A: We have removed the CFA analysis from the study due to the inadequacy of our sample size and the suggestion of the second reviewer.

4: **Lastly, factors should be retained using Parallel Analysis. Scree plot and Eigen values are traditional methods**

A: This had been done thank you very much for the great inputs to our manuscript (Page 10, line 219).

**The discussion part of this paper is not good. It would be better if you could write this part focusing on the results from the study**

A: We have overhauled the discussion section as recommended. Thank you.

Reviewer #2: It seems that the translation and adaptation steps as well as the validity and reliability of the questionnaire have been done to an acceptable level. However, there are problems that should be addressed by the authors:

**- In the first principal component analysis, three factors with a variance accounting for 62% were obtained. What was the authors' justification for the second principal component analysis with two factors and less variance coverage than the previous one (about 51%)? (lines: 247-283]**

A: thank you very much for this important observation. We wanted a structure that could be explainable looking at the items loading in them. However we have decided to stick to the initial PCA analysis as it has more variance coverage and the factors can also be explained.

**- Principal component analysis for construct validity was not performed in a single-factor model but was performed in confirmatory factor analysis; What is the reason for this.**

A: Thank you very much. Events have overridden this as we have removed the CFA in its entirety from the study.

**- The amount reported to cover the variance of the second principal component analysis in the abstract (51.7%) does not match with main text (51.47%).**

A: Thank you very much for noting this error. We have however expunged the second rotation and stuck to the first.

**- Report the factor loading of all items in the exploratory factor analysis output table**

A: We have reported the factor loadings of all items in the exploratory factor analysis output table (Page 14, 291).

- **The population under study in exploratory and confirmatory factor analysis should be distinct. However, none of the model versions had the optimal fit indices in factor analysis. Therefore, it may be advisable not to perform a factor analysis in this article and perform it in a distinct population.**

A: thank you very much for the advice, this partly informed our removal of the CFA from our analysis

**- The authors in the results section in the abstract have stated that: " the ODI-Y yielded a two-factor structure which accounted for 51.7% of the 54 variance but showed poor fit."
But in the discussion section they stated that: "the ODI was adapted into the Yoruba language and proved to have a 58 good factor structure and psychometric properties that replicated the results of other 59 obtainable versions." Don't these two terms contradict each other?**

 A: Thank you very much. This was an error and we have adjusted the write up only to highlight the psychometric properties what were explored in the study.
